# Redefining High-Risk and Mobile Population in Pakistan Polio Eradication Program; 2024

**DOI:** 10.3390/vaccines13101016

**Published:** 2025-09-29

**Authors:** Irshad Ali Sodhar, Jaishri Mehraj, Anum S. Hussaini, Shabbir Ahmed, Ahmed Ali Shaikh, Asif Ali Zardari, Sundeep Sahitia, Shumaila Rasool, Azeem Khowaja, Erin M. Stuckey

**Affiliations:** 1 Emergency Operation Center Sindh, Government of Sindh, Karachi 75510, Pakistan; 2 Integral Global Health Inc., Islamabad 44000, Pakistan; jaishrimehraj@gmail.com; 3 Harvard T.H. Chan School of Public Health, Boston, MA 02115, USA; anum.shussaini@gmail.com; 4 World Health Organization (WHO), Islamabad 45500, Pakistan; sahmed@who.int (S.A.); asifa@who.int (A.A.Z.); sahitias@who.int (S.S.); 5 Riz Consulting, Islamabad 44000, Pakistan; ahmed.ali@rizconsulting.biz; 6 National Stop Transmission of Polio (N-STOP) Program, Karachi 75510, Pakistan; drshumailazubair@gmail.com; 7 United Nations International Children’s Emergency Fund (UNICEF), Islamabad 44050, Pakistan; akhowaja@unicef.org; 8 Bill & Melinda Gates Foundation, Seattle, WA 98109, USA; erin.stuckey@gmail.com

**Keywords:** Pakistan, polio, vaccination, public health, immunization, high-risk populations, Sindh

## Abstract

Background: This study aimed to analyze the patterns and underlying reasons associated with population movement across Sindh, Pakistan. Methods: Cross-sectional surveys were conducted in response to the detection of WPV1 in various districts in Sindh province, where genetic linkages with poliovirus isolates in Karachi had been identified. The surveys targeted union councils (UCs) contributing sewage to the environmental sample collection sites where WPV1 was detected. Results: In the Karachi division a total of 1392 participants were interviewed, and outside Karachi 1471 participants were included. A significantly higher proportion of female participants were interviewed in Karachi (*n* = 72, 55.0%) compared to other divisions of Sindh (*n* = 794, 45.0%) (*p* < 0.001). Linguistic distribution varied significantly between regions, with Pashto speakers predominating in Karachi (*n* = 336, 86.4%), and Sindhi in other divisions (*n* = 501, 79.4%) (*p* < 0.001). OPV coverage exceeded 90% across all districts, and over 85% of children received RI vaccines. Travel patterns also differed significantly; participants from Karachi (*n* = 686, 44.2%) were less likely to report travel compared to other divisions (*n* = 865, 55.8%), who frequently traveled for family events, business, or employment (*p* < 0.001). Conclusions: It is critical to redefine high-risk populations annually based on updated mobility data, social survey analyses, and virus detection via surveillance to better identify and reach unvaccinated children in the Pakistan polio program. In addition, strategically placed PTPs along both formal and informal travel corridors based on an updated risk framework will enhance vaccination, thereby reducing the risk of virus spread.

## 1. Introduction

Wild poliovirus (WPV) infection, particularly poliomyelitis, disproportionately affects children under five, leading to paralysis and significant long-term consequences for both the children and their families [1]. In Pakistan, those most affected by polio often reside in low-income, marginalized communities where health infrastructure, water, and sanitation systems are inadequate. These children are also more likely to be under- or unvaccinated against routine antigens, including polio. Since the launch of the Global Polio Eradication Initiative (GPEI) in 1988, global polio cases have decreased by more than 99%, with only 12 cases reported worldwide in 2023, primarily in Afghanistan and Pakistan. In response to the ongoing transmission of WPV, Pakistan declared polio a national emergency in 1994. It continues to prioritize its polio eradication program through emergency operations centers (EOCs) at national and provincial levels [2]. The EOC network, led by the government of Pakistan, serves as a coordinating body for GPEI partners, including the US Centers for Disease Control and Prevention (CDC), UNICEF, the WHO, Rotary International, the Bill and Melinda Gates Foundation, and Gavi, the Vaccine Alliance (GAVI) [3].

Key programmatic elements of Pakistan’s polio eradication strategy include routine immunization (RI), supplementary immunization activities (SIAs) with the oral polio vaccine (OPV), and the inactivated polio vaccine (IPV). Surveillance measures target acute flaccid paralysis (AFP) in children and environmental monitoring to detect poliovirus in sewage. This multi-tiered approach is bolstered by community engagement and communication efforts to ensure widespread vaccine acceptance. The national Expanded Program on Immunizations (EPIs) in Pakistan was introduced in 1978, targeting routine immunization (RI) vaccines against six diseases, including polio [4]. These services are provided at fixed immunization centers and some outreach vaccination at different locations. Supplementary immunization activities (SIAs), including national and sub-national immunization activities (NIDs and SNIDs), are the main strategies to enhance OPV coverage besides four doses of routine OPV within RI services [5]. The Pakistan National Polio Program coordinates SIAs designed to enhance polio vaccine coverage, especially in high-risk populations. These campaigns typically span 5 to 7 days, during which vaccinators aim to reach every household with children under the age of five. In the initial 5 days, vaccinators visit all targeted households, while the remaining 2 days are dedicated to revisiting homes where children were either unavailable or refused vaccination during the first attempt [6]. NIDs are countrywide mass immunization drives, whereas SNIDs target high-priority districts or areas with greater vulnerability to poliovirus transmission [7]. To address cross-border poliovirus transmission, the Pakistan Polio Eradication Program has also established permanent transit points (PTPs) at key locations, such as the Pakistan–Afghanistan border, and interprovince or inter-district borders, ensuring that all travelers are vaccinated before entering or leaving the country [8].

Given that only approximately 1 in 200 poliovirus infections results in paralysis, the Pakistan Polio Eradication Program employs environmental surveillance as a critical tool for early detection of viral circulation. Samples are collected from sewage sites in various regions using established methods to detect the presence of poliovirus in the population before it causes paralysis [9,10]. This strategy enables health officials to promptly launch targeted vaccination campaigns, aiming for sufficiently high immunization coverage to interrupt transmission before widespread outbreaks occur. Through this proactive surveillance, the program can identify areas with potential viral spread, allowing for more efficient and focused public health interventions. A regional reference laboratory for poliomyelitis at the National Institute for Health (NIH), Islamabad, Pakistan, conducts investigations into the genetic relatedness of poliovirus isolates [11]. The WHO-recommended procedures for detecting and characterizing polioviruses from the stool samples of AFP cases and sewage water samples collected from the environment are followed by the regional reference laboratory for polio [10,11]. Within the polio program network of Pakistan, reports of WPV1 detection from any sample are shared internally [10].

Population mobility plays a critical role in the transmission of WPV, especially across district borders within Sindh and between Pakistan and neighboring Afghanistan. Unregulated population movement, especially through informal routes, has been linked to WPV being reintroduced into districts previously free of the virus. In 2024, population movement within Sindh intensified due to socioeconomic drivers, increasing the risk of viral spread. A study conducted across various districts in Sindh showed that these population movements often occurred amongst populations with low immunization rates, creating pockets of vulnerable children susceptible to infection. Karachi and other urban centers have seen a marked increase in WPV cases due to high internal migration and transient populations [12]. Moreover, population movements within Sindh have been directly linked to the spread of poliovirus. Seasonal labor migration, particularly to and from agricultural districts such as Kambar, Sukkur, Thatta, and Badin, has been identified as a significant driver of virus transmission. These movements complicate efforts to maintain high vaccine coverage as mobile populations are often missed during routine immunization campaigns.

This study aimed to identify the patterns of movement of the population within different districts of Sindh province and to determine the reasons associated with the extensive population movement within different districts of Sindh, Pakistan, to discover potential links between travel from WPV1-infected areas and the occurrence of poliovirus within the studied population.

## 2. Materials and Methods

### 2.1. Study Design, Settings, and Population

Cross-sectional surveys were conducted in various districts within the Karachi division and other divisions of Sindh, Pakistan, in response to the detection of WPV1 in the environmental surveillance (ES) sites of various districts in Sindh province that have genetic linkages with strains isolated in Karachi. The surveys targeted union councils (UCs) contributing sewage to the ES collection sites where WPV1 was identified. Within Karachi, surveys were conducted in the Central, Keamari, Malir, South, and West districts. In other Sindh divisions, surveys included the districts of Badin, Hyderabad, Kambar, Mirpurkhas, SBA, Sujawal, and Sukkur. Later, the surveys were also conducted in response to human polio cases reported in Sindh. Details regarding WPV1 isolation, genetic linkages, and survey timelines are outlined below.

On 7 March 2024, an environmental sample collected from the Mahwali Dargah Nalla site in the Badin district tested positive for WPV1. Genetic sequencing revealed its linkage to a WPV1 strain detected in an ES sample from Haji Mureed Goth in Karachi Central, collected on 15 February 2024. Similarly, on 4 March 2024, an ES sample from the Tulsidas Pumping Station in Hyderabad district tested positive for WPV1, genetically linked to the same Haji Mureed Goth strain. In response, the polio emergency operations center (PEOC) Sindh conducted population movement tracking surveys in April 2024 across the affected districts of Badin, Hyderabad, and Karachi Central. Surveys focused on 19 UCs contributing sewage to the affected ES sites. Subsequently, an ES sample from the Ring Road Puraan site in Mirpurkhas district, collected on 2 April 2024, tested positive for WPV1. Genetic analysis linked this strain to a virus detected in an ES sample from Orangi Nalla in Keamari, Karachi, collected on 4 March 2024. Additionally, an ES sample collected on 2 April 2024 from the Maka Pumping Station in the Sukkur district also tested positive for WPV1, with a 99.77% genetic match to a virus detected in Karachi Central on 2 January 2024. In May 2024, PEOC Sindh conducted population movement tracking surveys in Karachi Central, Keamari, Sukkur, Mirpurkhas, and Karachi West to address these findings.

A WPV1-confirmed human case was reported on 13 May 2024 from UC Bhirkan, Tehsil Lakhi, District Shikarpur, Sindh Province. Genetic sequencing identified the strain as part of the YB3A cluster, with a 99.6% match to an ES sample collected on 12 March 2024 from Karachi South. The case had a travel history to Malir, Karachi, and the family hosted guests in March from Bhens Colony, Malir. PEOC Sindh responded by conducting movement-tracking surveys in Malir, Karachi, in June 2024.

Another WPV1-confirmed human case was reported on 5 June 2024 from UC Keamari 3, Karachi, Keamari district. This case’s virus strain, also from the YB3A cluster, showed a 99.89% genetic match to an ES sample collected from the Orangi Nalla site, Karachi, Keamari, on 4 March 2024. Movement tracking surveys were conducted in July 2024 in the South Karachi, Keamari, and Kambar districts to address these findings.

In August 2024, a WPV1 human case was reported in the Hyderabad district, linked to an ES sample from Hyderabad. Furthermore, ES samples from Shaheed Benazirabad (Jamshed Colony main disposal site) and Sujawal (Haqabad Pumping Station), collected on 6 August 2024, tested positive for WPV1. The SBA sample showed a 99.55% genetic match with an ES isolate from Okara (collected on 19 February 2024), while the Sujawal sample had a 99.66% match with an ES isolate from Hyderabad (collected on 8 May 2024). In response, PEOC Sindh conducted movement-tracking surveys in the Sujawal, Shaheed Benazirabad, and Hyderabad districts during August 2024. Supplementary Table S1 shows the sequence of the surveys conducted in response to different events of WPV1 occurrence at different time points in Sindh. The detailed questionnaire used in this study is also provided in Supplementary Materials.

### 2.2. Study Variables and Data Collection

Information was collected through structured interviews with the adult participants. Socio-demographic data included participant age, gender, language, tribal affiliation, number of children under five years old, number of children under two years old, recent guest arrivals (within the last three months), and recent travel to any district within Pakistan. Additional information included the participant’s recent interactions with polio teams, focusing on team behavior towards the community, vaccination status of children under five years old, vaccination status of guest children, travel purpose, travel frequency, and household income sources. Data collection was conducted by UC-level polio program staff, with supervision provided by designated districts and provincial monitors.

### 2.3. Data Management and Analysis

Survey data were collected via an online Google Form, and the resulting datasets were exported to Microsoft Excel for preprocessing before analysis in IBM SPSS Statistics version 22. Descriptive statistics were used to summarize the data. Categorical variables (e.g., gender, language, oral polio vaccination status, and routine immunization status) were reported as frequencies and proportions, while continuous variables (e.g., respondent age and the number of children under five years old) were reported as means with standard deviation (SD). Inferential statistical analyses were performed to identify significant differences between groups. The Pearson Chi-square test assessed associations between binary and categorical variables in the Karachi division and other Sindh divisions. Independent sample *t*-tests were conducted to evaluate differences in continuous variables between these geographic divisions.

### 2.4. Ethical Considerations

Ethical approval for the study was obtained from the Provincial Bioethics Committee of the Director General Health Services, Government of Sindh. Study procedures were thoroughly explained to all participants, and informed consent was obtained before data collection. To ensure anonymity, personal identifiers, such as participant names, were excluded from the administered questionnaires. Data collected during the survey were exclusively used for research purposes, with strict prohibitions on unauthorized reproduction or distribution.

## 3. Results

### 3.1. Participant Characteristics in the Karachi Division

In the Karachi division, a total of 1392 participants were interviewed, distributed across the districts as follows: 636 (45.7%) from Central, 240 (17.2%) from Malir, 231 (16.6%) from Keamari, and 225 (16.2%) from the West and 60 (4.3%) from the South districts of the Karachi division.

The participants’ mean age was 34.17 years (standard deviation = 9.51), ranging from 18 to 81 years. Most of the respondents were female (972; 69.9%). The linguistic diversity among participants included the following: 81 (5.8%) Balochi speakers, 336 (24.1%) Pashto, 155 (11.1%) Punjabi, 130 (9.3%) Sindhi, 118 (8.5%) Saraiki, 376 (27.0%) Urdu, and 196 (14.1%) speaking other languages.

Households reported having between 0 and 9 children under five years of age. Of these children, 1310 (94.1%) received OPV during the last campaign. OPV coverage exceeded 90% across all districts, and over 85% of children were reported to have received RI vaccines. Among respondents, 917 (65.9%) reported their children had completed the full routine immunization schedule, 344 (24.7%) reported partial immunization, and 131 (9.4%) stated their children had not received any routine vaccination.

Travel behaviors revealed that 686 (49.3%) participants reported at least one household member traveling outside Karachi within the last six months, while 1058 (75.9%) hosted guests during the same period. Of these, 364 (26.1%) reported that guest children were vaccinated in their households. Over 70% of participants reported guest arrivals across all districts in the Karachi division.

Regarding polio vaccination visibility, 703 (50.5%) participants observed polio vaccination activities within the city, while 553 (39.7%) witnessed such activities outside Karachi. The primary reasons for travel were attending family events (245; 17.6%), followed by job-related travel (50; 3.6%) and religious events (38; 2.7%). Less than 1% of participants traveled for business or educational purposes, and 121 (8.7%) did not disclose their reasons for travel.

A total of 256 (18.4%) participants reported dual residence (houses in multiple districts). Among those, the primary reasons for visiting their hometowns included family events (365; 26.2%), Eid holidays (67; 4.8%), monthly visits (44; 3.2%), summer holidays (32; 2.3%), and weekly visits (26; 1.9%). However, 301 (21.6%) participants did not disclose the frequency of such visits.

Employment emerged as a significant factor for residents in Karachi, as reported by 510 (36.6%) participants, followed by business (183; 13.2%). Employment was also identified as the primary source of household income with 515 (37.1%) respondents. Table 1 presents a detailed overview of the socio-demographic and travel characteristics of the study participants across the five districts of the Karachi division, Sindh, Pakistan.

### 3.2. Participant Characteristics in Other Divisions of Sindh Province

Surveys were conducted across various districts in Sindh outside the Karachi division, encompassing 1471 participants. The distribution of participants by district was as follows: 146 (9.9%) from Badin, 498 (33.9%) from Hyderabad, 136 (9.2%) from Kambar, 228 (15.5%) from Mirpurkhas, 89 (6.1%) from SBA (Shaheed Benazirabad), 60 (4.1%) from Sujawal, and 314 (21.3%) from Sukkur.

The proportion of females in these districts was 749 (54%), though Kambar and Sujawal reported a lower female-to-male ratio. Sukkur and Mirpurkhas exhibited linguistic diversity, with 43% of participants speaking Urdu, 34% Sindhi, and 6.7% speaking other languages. The OPV vaccination coverage was high at 1422 (96.7%) overall. However, small gaps in coverage were observed, with 15 (3%) participants in Hyderabad and 7 (3.1%) in Mirpurkhas reporting unvaccinated children. RI vaccination completion rates were 1035 (70.4%), while 238 (16.2%) were partially vaccinated. District-specific analysis revealed that Sukkur (21.3%), Kambar (24%), Hyderabad (11.8%), and Mirpurkhas (11%) had notable proportions of children who were not vaccinated with any RI antigen. Partial immunization rates were highest in Mirpurkhas (44.7%) and Sukkur (29.3%).

**Figure 1:**
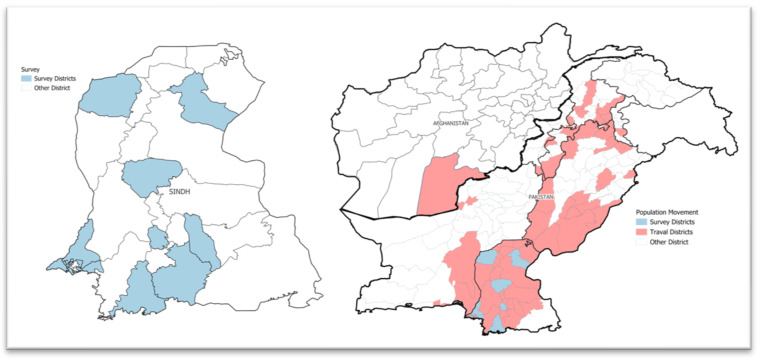
The scope of districts where surveys were conducted and the travel history of survey participants within different districts of Pakistan.

Overall, guest arrivals were reported by 1094 (74.4%) participants. Hyderabad (89.6%), Sujawal (80.0%), and Badin (76.7%) had the highest proportions of guest arrivals, while SBA reported a 48.3% non-arrival rate. Travel outside the districts was reported by 865 (58.8%) participants, with the highest travel frequency observed in Sujawal (83.3%), Badin (72.6%), and Hyderabad (68.1%). Most travel was attributed to attending family events. Figure 1 shows the districts where surveys were conducted and the travel of survey participants to different districts of Pakistan. Dual residence or having houses in another district was reported by 230 (15.6%) participants. Visits to hometowns were primarily for family events (190; 12.9%), followed by business (65; 4.4%), jobs (37; 2.5%), religious events (24; 1.6%), and education (1; 0.1%). An additional 163 (11.1%) participants cited unspecified reasons for their travel. Employment was a primary reason for residence in these districts for 37% of participants, followed by business (13%). Employment was also the main source of income for 19% of participants in the Karachi division. Table 2 provides a detailed breakdown of the socio-demographic and travel characteristics of study participants in the outbreak districts of other divisions of Sindh province, Pakistan.

### 3.3. Binary Analysis: Comparison Between Karachi Division and Other Divisions of Sindh

Binary analysis highlights significant differences between survey participants from the Karachi division and other divisions of Sindh. Key findings show that a significantly higher proportion of females participated in Karachi (72; 55.0%) compared to other divisions of Sindh (794; 45.0%) (Chi-square *p*-value < 0.001). Pashto speakers predominated in Karachi with 336 (86.4%) participants, whereas Sindhi speakers were the majority in other divisions, accounting for 501 (79.4%) (Chi-square *p*-value < 0.001).

Differences in OPV coverage were statistically significant (*p*-value < 0.001). Unvaccinated children were more prevalent in other divisions (198; 60.2%), while partially vaccinated children were more common in Karachi (344; 59.1%) (Chi-square *p*-value < 0.001). Statistically significant differences were observed in the vaccination rates of guest children between Karachi and other divisions (Chi-square *p*-value < 0.001).

Travel history and the purpose of travel varied significantly between the two regions. Participants from Karachi were less likely to report travel outside the city, while those in other divisions often traveled for family events, business, or jobs (Chi-square *p*-value < 0.001). Employment was a key source of income in Karachi, whereas other divisions reported diverse income sources, including business and agriculture. This difference was statistically significant (Chi-square *p*-value < 0.001).

Table 3 provides a detailed statistical comparison of these characteristics, underscoring the socio-demographic, vaccination, and behavioral differences between participants in the outbreak districts of Karachi and the other divisions of Sindh province, Pakistan.

## 4. Discussion

This study identified frequent population movement within districts of Karachi and other divisions of Sindh, with the primary reason for travel being attendance at family events. A high proportion of guest arrivals serves as secondary evidence of substantial population mobility. Additionally, a significant proportion of the population maintains a second residence in another district, where they frequently travel. Individuals with dual residences primarily reside in their current locations due to employment or business obligations. Among the participants surveyed, employment or formal/informal jobs were reported as the main source of income. Overall, optimal oral polio vaccine (OPV) coverage from SIAs and routine immunization were reported across most districts in Karachi and other divisions of Sindh. However, studies conducted over the past five years indicate that areas with high population mobility, particularly following natural disasters, remain critical to polio eradication efforts [13]. Population movement is evident through household member travel or guest arrivals. In this study, 49% of households in Karachi reported at least one member traveling outside the city, with the primary reasons for travel being family events (18%), employment (4%), and religious events (3%). Less than 1% traveled for business or educational reasons. In other divisions of Sindh, 59% of respondents reported travel outside their districts, with the highest travel frequencies observed in Sujawal (83%), Badin (73%), and Hyderabad (68%), primarily for family events. Additionally, 18% of participants in Karachi and 16% in other districts of Sindh reported maintaining a secondary residence in another district. The predominant reasons for visiting these hometowns included family events (26%) and employment (37%), followed by business (13%) in Karachi. In other divisions of Sindh, family visits (13%) remained the primary reason for travel, followed by business (4%), employment (3%), religious events (2%), and educational purposes (0.1%).

The province’s unique sociopolitical landscape is characterized by high rates of rural-to-urban migration and internal displacement that requires a frequent reassessment of population movement dynamics in addition to the known seasonal movement trends due to weather and employment. In the years leading up to this study, several major events have impacted population movement and shifted the polio risk landscape in the province. In 2022, flooding of the Indus River impacted 33 million people in 84 of the districts in Pakistan, and the impact was highest in Sindh as 70% of the country’s total losses and damages occurred in Sindh province, devastating health care, employment opportunities, and housing for people for over a year. In late 2023 and early 2024, there were Afghan repatriation actions conducted by the Pakistan interior ministry, which also spurred population movement both within Sindh and between other provinces in Pakistan. Additional region-specific population movement events within Sindh province have been described elsewhere [14].

The ongoing poliovirus transmission, particularly in Sindh province, is attributed to a complex interplay of both seasonal and event-driven population movements, suboptimal immunization coverage, and challenges in accessing high-risk populations. Factors such as these complicate the delivery of both campaign and routine immunization services to vulnerable populations despite Sindh’s province-wide electronic immunization registry for routine immunization as it requires both the availability of services from the health care system and the motivation for parents to access services for their children. Surveys conducted in 2023 and 2024 across various districts in Sindh, including high-risk areas such as Karachi, Larkana, and Hyderabad, highlighted persistent gaps in immunization coverage despite targeted SIAs. Although overall immunization rates have improved, children in hard-to-reach and mobile populations, such as internally displaced persons, remain under-vaccinated, sustaining pockets of poliovirus transmission [13].

The Pakistan polio program has historically identified three major poliovirus reservoirs—Karachi, Peshawar, and the Quetta block—that account for a substantial proportion of WPV1 cases (64.2% in 2015, 75.4% in 2016, and 76.7% in 2017), as indicated by epidemiological analyses of WPV1 transmission [15,16]. The sustained poliovirus circulation in these reservoir areas is further exacerbated by periodic importations, driven by extensive population movement between these high-risk reservoirs and Afghanistan [14,17]. The Pakistan–Afghanistan polio program considers both countries as a single poliovirus reservoir due to the frequent cross-border movement, which facilitates the sharing of multiple poliovirus lineages [18,19]. A previous study found that overall, 84% of children originated outside the district of their current location, belonging to high-risk and mobile populations (HRMPs) with substantial links with Afghanistan and other regions of Pakistan [17]. Given this dynamic, the vaccination of children in the HRMP remains a high priority for the polio eradication program.

Achieving high vaccination coverage is crucial for interrupting poliovirus transmission and attaining global polio eradication goals. In this study, OPV coverage was reported at 90% in Karachi and 97% in other divisions of Sindh. Routine immunization coverage was over 85% in Karachi, with 66% of children fully vaccinated, 25% partially vaccinated, and 9% unvaccinated. In other divisions, routine immunization coverage was 70% fully vaccinated and 16% partially vaccinated. A previous study also reported 98% OPV coverage among children in the HRMP [17]. Nevertheless, deliberate overreporting of vaccination status by household members and recall bias must be considered as potential limitations. In this study, 49% of guest children were reportedly vaccinated by polio teams at their homes in Karachi, while 40% of guest children received polio vaccines in other divisions of Sindh. Earlier studies conducted in Karachi reported lower proportions of fully vaccinated children. This difference could have several explanations. It could indicate an improvement in the EPI program’s ability to access vulnerable children [14,20]. It is also possible that the proportion of fully immunized children was overestimated in our study, as detailed information on each EPI vaccine was not collected separately in the questionnaire. The overreporting of vaccination rates is a recognized phenomenon, and discrepancies between reported and verified coverage rates have been documented in prior studies [21,22]. Independent verification mechanisms are essential to validate reported data and ensure accurate assessments of vaccination status.

Furthermore, the detection of human-confirmed polio cases and polioviruses in environmental samples across Sindh, Pakistan, confirms the presence of unvaccinated or under-vaccinated children in all divisions. The frequent movement of these unimmunized or partially immunized populations may contribute to the introduction or reintroduction of the poliovirus into the local communities [14]. Additionally, poliovirus transmission may persist even among certain fully vaccinated but asymptomatic children. Suboptimal vaccine seroconversion, particularly in malnourished populations, along with factors such as overcrowding, high poliovirus concentrations in the environment, and competition with other enteric viruses, have been reported in previous studies from the region [23,24].

Given that infectious diseases such as polio are propagated through population movement [25], a comprehensive understanding of migration patterns is essential for developing targeted vaccination strategies and effectively interrupting poliovirus transmission.

Pakistan shares a long and porous border with Afghanistan—approximately 2600 km in length—across which there is substantial cross-border movement, both through formal border crossings and a multitude of informal routes. To effectively interrupt poliovirus transmission, especially in the context of population mobility, the following targeted measures are critical for Pakistan. Strengthening transit vaccination at formal and informal crossings; while vaccination at major formal transit points (e.g., Torkham and Chaman) exists, it must be expanded to include informal and less-monitored crossings. Establishing static vaccination sites at key informal routes can help reach populations that bypass official checkpoints. Comprehensive screening of all vehicles; transit vaccination efforts must extend beyond public transportation. It is essential to ensure that all modes of transport—including private vehicles, freight carriers, and pedestrian crossings—are screened and covered by vaccination teams. All-age polio vaccination strategy at borders; given the risk of transmission across age groups, vaccination efforts at border areas should include all age groups, not just children under five. This strategy aligns with the detection of virus excretion in older individuals and would contribute to interrupting silent transmission. Mapping and real-time monitoring of migration routes; a robust, dynamic system to map both seasonal and daily migration flows—covering nomadic populations, informal labor migrants, and cross-border traders—will enable a more responsive and strategic placement of vaccination teams. Bilateral coordination with Afghanistan; joint border health committees and synchronized vaccination campaigns on both sides of the border will ensure harmonized efforts and reduce the risk of re-importation of the virus.

## 5. Conclusions

This investigation highlights substantial population mobility observed in different districts of Sindh province in Pakistan. The results of this study indicate that the Sindh polio program requires frequent reassessment of population movement dynamics to redefine high-risk and mobile populations. A multifaceted approach is essential to identify and prioritize high-risk populations, particularly those frequently traveling through informal routes. Strengthening PTPs and strategically establishing additional PTPs along informal travel corridors will enhance the ability to vaccinate children on the move, thereby reducing the risk of virus spread.

While the polio eradication program has predominantly focused on Pashto-speaking populations from KP and Afghanistan, there is growing concern that missed vaccinations among Balochi-speaking populations may also contribute to the continued circulation of WPV1. To mitigate this risk, it is critical to redefine high-risk populations annually based not only on updated mobility data but also on social survey analyses. This dynamic, data-driven approach will enable the program to focus on populations with the greatest risk of WPV1 transmission and ensure more equitable vaccine coverage.

Currently, the program categorizes missed children as not available or refusing. However, a more granular analysis is required to identify the specific characteristics of these missed children and the population subgroups to which they belong. A targeted immunization strategy, informed by such analysis, will help improve the immunity profile of the most vulnerable populations.

Environmental surveillance serves as an early indicator of WPV1 circulation, often detecting viruses in wastewater before human cases emerge. Given its predictive value, we recommend that the polio eradication program redefine high-risk populations annually based on ES data or outbreak occurrences. Additionally, integrating the mandatory tracking of population movement through social surveys will provide critical insights into mobility patterns, enabling a proactive approach to identifying and vaccinating populations at a heightened risk of poliovirus exposure. A sustained and adaptive strategy, informed by real-time data on population mobility and immunization gaps, will be pivotal in achieving polio eradication in Pakistan.

## Figures and Tables

**Table 1:** Characteristics of study participants in five districts of Karachi division of Sindh, Pakistan.

Variable	Central	Keamari	Malir	South	West	Karachi
	(*N* = 636)	(*N* = 231)	(*N* = 240)	(*N* = 60)	(*N* = 225)	(*N* = 1392)
**Gender**						
Female	480 (75.5%)	152 (65.8%)	103 (42.9%)	50 (83.3%)	187 (83.1%)	972 (69.9%)
Male	156 (24.4%)	79 (34.2%)	137 (57.1%)	10 (16.7%)	38 (16.9%)	420 (30.2%)
**Age** (mean ± SD)	33.364 (9.2543)	34.872 (8.6133)	34.963 (10.2877)	35.317 (8.8825)	34.576 (10.2688)	34.170 (9.5103)
**Language**						
Balochi	18 (2.8%)	16 (6.9%)	5 (2.1%)	19 (31.7%)	23 (10.2%)	81 (5.8%)
Pashto	107 (16.8%)	121 (52.4%)	19 (7.9%)	0 (0.0%)	89 (39.6%)	336 (24.1%)
Punjabi	68 (10.7%)	13 (5.6%)	64 (26.7%)	4 (6.7%)	6 (2.7%)	155 (11.1%)
Sindhi	61 (9.6%)	11 (4.8%)	49 (20.4%)	5 (8.3%)	4 (1.8%)	130 (9.3%)
Siraiki	96 (15.1%)	6 (2.6%)	15 (6.3%)	0 (0.0%)	1 (0.4%)	118 (8.5%)
Urdu	180 (28.3%)	17 (7.4%)	67 (27.9%)	23 (38.3%)	89 (39.6%)	376 (27.0%)
Others	106 (16.7%)	47 (20.3%)	21 (8.8%)	9 (15.0%)	13 (5.8%)	196 (14.1%)
**Number of children under five years of age** (mean ± SD)	1.602 (0.8734)	1.632 (0.8840)	1.533 (0.9545)	1.617 (0.8456)	1.622 (1.3108)	1.599 (0.9706)
**OPV vaccination in SIA status**						
No response	5 (0.8%)	1 (0.4%)	5 (2.1%)	1 (1.7%)	3 (1.3%)	15 (1.1%)
No	27 (4.2%)	9 (3.9%)	10 (4.2%)	3 (5.0%)	18 (8.0%)	67 (4.8%)
Yes	604 (95.0%)	221 (95.7%)	225 (93.8%)	56 (93.3%)	204 (90.7%)	1310 (94.1%)
**RI status**						
No	55 (8.6%)	11 (4.8%)	30 (12.5%)	3 (5.0%)	32 (14.2%)	131 (9.4%)
Yes partial	144 (22.6%)	67 (29.0%)	60 (25.0%)	6 (10.0%)	67 (29.8%)	344 (24.7%)
Yes complete	437 (68.7%)	153 (66.2%)	150 (62.5%)	51 (85.0%)	126 (56.0%)	917 (65.9%)
**Guest arrival**						
No	147 (23.1%)	66 (28.6%)	56 (23.3%)	16 (26.7%)	49 (21.8%)	334 (24.0%)
Yes	489 (76.9%)	165 (71.4%)	184 (76.7%)	44 (73.3%)	176 (78.2%)	1058 (75.9%)
**Any Guest children vaccinated**						
No response	224 (35.2%)	27(11.7%)	56 (23.3%)	16 (26.7%)	22 (9.8%)	345 (24.8%)
No	177 (27.8%)	50 (21.6%)	54 (22.5%)	21 (35.0%)	62 (27.6%)	364 (26.1%)
Yes	235 (36.9%)	154 (66.7%)	130 (54.2%)	23 (38.3%)	141 (62.7%)	683 (49.1%)
**Travel history**						
No	346 (54.4%)	91 (39.4%)	120 (50.0%)	39 (65.0%)	110 (48.9%)	706 (50.7%)
Yes	290 (45.6%)	140 (60.6%)	120 (50.0%)	21 (35.0%)	115 (51.1%)	686 (49.3%)
**Purpose of travel**						
Business	0 (0%)	1 (0.4%)	3 (1.3%)	0 (0.0%)	1 (0.4%)	5 (0.4%)
Education	0 (0%)	0 (0.0%)	1 (0.4%)	0 (0.0%)	0 (0.0%)	1 (0.1%)
Job	12 (1.9%)	8 (3.5%)	28 (11.7%)	0 (0.0%)	2 (0.9%)	50 (3.6%)
Family event	82 (12.9%)	56 (24.2%)	48 (20.0%)	4 (6.7%)	55 (24.4%)	245 (17.6%)
Religious event	8 (1.3%)	19 (8.2%)	6 (2.5%)	1 (1.7%)	4 (1.8%)	38 (2.7%)
Other	26 (4.1%)	15 (6.5%)	30 (12.5%)	2 (3.3%)	48 (21.3%)	121 (8.7%)
No information	162 (25.5%)	41 (17.7%)	4 (1.7%)	14 (23.3%)	5 (2.2%)	226 (16.2%)
NA	346 (54.4%)	91 (39.4%)	120 (50.0%)	39 (65.0%)	110 (48.9%)	706 (50.7%)
**Witnessing polio vaccination at another location in the City**						
No	340 (53.5%)	102 (44.2%)	128 (53.3%)	46 (76.7%)	73 (32.4%)	689 (49.5%)
Yes	296 (46.5%)	129 (55.8%)	112 (46.7%)	14 (23.3%)	152 (67.6%)	703 (50.5%)
**Witnessing polio vaccination at another location outside the City**						
No	368 (57.9%)	145 (62.8%)	158 (65.8%)	54 (90.0%)	114 (50.7%)	839 (60.3%)
Yes	268 (42.1%)	86 (37.2%)	82 (34.2%)	6 (10.0%)	111 (49.3%)	553 (39.7%)
**Dual Houses**						
No	472 (74.2%)	214 (92.6%)	199 (82.9%)	52 (86.7%)	199 (88.4%)	1136 (81.6%)
Yes	164 (25.8%)	17 (7.4%)	41 (17.1%)	8 (13.3%)	26 (11.6%)	256 (18.4%)
**Frequency of visit to hometown**						
Every weekend	7 (1.1%)	2 (0.9%)	2 (0.8%)	10 (16.7%)	5(2.2%)	26 (1.9%)
Every month	17 (2.7%)	3 (1.3%)	5 (2.1%)	9 (15.0%)	10 (4.4%)	44 (3.2%)
On Eid holidays	32 (5.0%)	14 (6.1%)	16 (6.7%)	1 (1.7%)	4 (1.8%)	67 (4.8%)
Summer holidays	14 (2.2%)	8 (3.5%)	5 (2.1%)	5 (8.3%)	0 (0.0%)	32 (2.3%)
Family events	205 (32.2%)	47 (20.3%)	56 (23.3%)	24 (40.0%)	33 (14.7%)	365 (26.2%)
Other	110 (17.3%)	21 (9.1%)	75 (31.3%)	10 (16.7%)	85 (37.8%)	301 (21.6%)
No information	251 (39.4%)	136 (58.8%)	81 (33.8%)	1 (1.7%)	88 (39.1%)	557 (40.0%)
**Purpose of staying here**						
Business	26 (4.1%)	53 (22.9%)	43 (17.9%)	2 (3.3%)	59 (26.2%)	183 (13.2%)
Education	9 (1.4%)	1 (0.4%)	6 (2.5%)	4 (6.7%)	3 (1.3%)	23 (1.7%)
Family event	7 (1.1%)	6 (2.6%)	6 (2.5%)	0 (0.0%)	3 (1.3%)	22 (1.6%)
Job	143 (22.5%)	79 (34.2%)	136 (56.7%)	51 (85.0%)	101 (44.9%)	510 (36.6%)
Other	30 (4.7%)	8 (3.5%)	12 (5.0%)	1 (1.7%)	18 (8.0%)	69 (5.0%)
No information	421 (66.1%)	84 (36.4%)	37 (15.4%)	2 (3.3%)	41 (18.2%)	585 (42.0%)
**Source of income**						
Business	0 (0.0%)	38 (16.4%)	39 (16.3%)	6 (10.0%)	0 (0.0%)	83 (6.0%)
Farmer	0 (0.0%)	1 (0.4%)	3 (1.3%)	8 (13.3%)	0 (0.0%)	12 (0.8%)
Govt. job	0 (0.0%)	8 (3.5%)	11 (4.6%)	1(1.7%)	0 (0.0%)	20 (1.5%)
Job	143 (22.5%)	9 (3.9%)	3 (1.3%)	1(1.7%)	101 (44.9%)	257 (18.5%)
Private job	0 (0.0%)	66 (28.6%)	134 (55.8%)	38 (63.3%)	0 (0.0%)	238 (17.1%)
Shopkeeper	0 (0.0%)	11 (4.8%)	9 (3.8%)	4 (6.7%)	0(0.0%)	24 (1.7%)
Other	0 (0.0%)	45 (195%)	28 (11.7%)	2 (3.3%)	0(0.0%)	75 (5.4%)
No information	493 (77.4%)	53 (22.9%)	13 (5.4%)	0 (0.0%)	124 (55.1%)	683 (49.1%)

Number indicates one response per household and its percentage from the total participants in that district.

**Table 2:** Characteristics of study participants in other outbreak districts of different divisions of Sindh, Pakistan.

Variable	Badin	Hyderabad	Kambar	Mirpurkhas	SBA	Sujawal	Sukkur	Sindh
	(*N* = 146)	(*N* = 498)	(*N* = 136)	(*N* = 228)	(*N* = 89)	(*N* = 60)	(*N* = 314)	(*N* = 1471)
**Gender**								
Female	102 (69.9%)	313 (62.9%)	10 (7.4%)	127 (55.7%)	26 (29.2%)	11 (18.3%)	205 (65.3%)	749 (54.0%)
Male	44 (30.1%)	184 (36.9%)	126 (92.6%)	100 (43.9%)	63 (70.8%)	49 (81.7%)	109 (34.7%)	675 (45.9%)
**Age** (mean ± SD)	38.838 (10.9597)	35.192 (7.7780)	36.978 (7.7378)	34.119 (10.4921)	36.865 (9.9569)	33.750 (8.0478)	36.965 (9.7752)	35.973 (9.2622)
**Language**								
Balochi	2 (1.4%)	0 (0.0%)	0 (0.0%)	25 (11.0%)	15 (16.9%)	0 (0.0%)	38 (12.1%)	80 (5.4%)
Pashto	20 (13.7%)	1 (0.2%)	0 (0.0%)	1 (0.4%)	0 (0.0%)	0 (0.0%)	31 (9.9%)	53 (3.6%)
Punjabi	12 (8.2%)	13 (2.6%)	0 (0.0%)	18 (7.9%)	11 (12.4%)	0 (0.0%)	3 (1.0%)	57 (3.9%)
Sindhi	90 (61.6%)	68 (13.7%)	96 (70.6%)	106 (46.5%)	6 (6.7%)	58 (96.7%)	77 (24.5%)	501 (34.1%)
Siraiki	0 (0.0%)	2 (0.4%)	1 (0.7%)	2 (0.1%)	16 (18.0%)	0 (0.0%)	28 (8.9%)	49 (3.3%)
Urdu	22 (15.1%)	412 (82.7%)	0 (0.0%)	64 (28.1%)	38 (42.7%)	0 (0.0%)	97 (30.9%)	633 (43.0%)
Others	0 (0.0%)	2 (0.4%)	39 (28.7%)	12 (5.3%)	3 (3.4%)	2 (3.3%)	40 (12.7%)	98 (6.7%)
**Number of children under five years of age** (mean ± SD)	1.589 (0.9371)	1.596 (0.7922)	2.213 (1.0357)	1.732 (0.9020)	1.708 (0.9909)	1.800 (0.9881)	2.226 (1.5407)	1.823 (1.0955)
**OPV vaccination in SIA status**								
No response	4 (2.7%)	2 (0.4%)	0 (0.0%)	5 (2.2%)	2 (2.2%)	0 (0.0%)	0 (0.0%)	13 (0.9%)
No	2 (1.4%)	15 (3.0%)	3 (2.2%)	7 (3.1%)	2 (2.2%)	2 (3.3%)	5 (1.6%)	36 (2.4%)
Yes	140 (95.9%)	481 (96.6%)	133 (97.8%)	216 (94.7%)	85 (95.5%)	58 (96.7%)	309 (98.4%)	1422 (96.7%)
**RI status**								
No	6 (4.1%)	59 (11.8%)	33 (24.3%)	25 (11.0%)	5 (5.6%)	3 (5.0%)	67 (21.3%)	198 (13.5%)
Yes partial	14 (9.6%)	30 (6.0%)	0 (0.0%)	102 (44.7%)	0 (0.0%)	0 (0.0%)	92 (29.3%)	238 (16.2%)
Yes complete	126 (86.3%)	409 (82.1%)	103 (75.7%)	101 (44.3%)	84 (94.4%)	57 (95.0%)	155 (49.4%)	1035 (70.4%)
**Guest arrival**								
No	34 (23.3%)	52 (10.4%)	62 (45.6%)	79 (34.6%)	43 (48.3%)	12 (20.0%)	95 (30.3%)	377 (25.6%)
Yes	112 (76.7%)	446 (89.6%)	74 (54.4%)	149 (65.4%)	46 (51.7%)	48 (80.0%)	219 (69.7%)	1094 (74.4%)
**Any Guest children vaccinated**								
No response	10 (6.8%)	89 (17.9%)	118 (86.8%)	119 (52.2%)	73 (82.0%)	42 (70.0%)	172 (54.8%)	623 (42.4%)
No	120 (82.2%)	23 (4.6%)	0 (0.0%)	52 (22.8%)	3 (3.4%)	1 (1.7%)	67 (21.3%)	266 (18.1%)
Yes	16 (11.0%)	386 (77.5%)	18 (13.2%)	57 (25.0%)	13 (14.6%)	17 (28.3%)	75 (23.9%)	582 (39.6%)
**Travel history**								
No	40 (27.4%)	159 (31.9%)	123 (90.4%)	123 (53.9%)	31 (34.8%)	10 (16.7%)	120 (38.2%)	606 (41.2%)
Yes	106 (72.6%)	339 (68.1%)	13 (9.6%)	105 (46.1%)	58 (65.2%)	50 (83.3%)	194 (61.8%)	865 (58.8%)
**Purpose of travel**								
Business	0 (0.0%)	10 (2.0%)	0 (0.0%)	14 (6.1%)	5 (5.6%)	5 (8.3%)	31 (9.9%)	65 (4.4%)
Education	0 (0.0%)	1 (0.2%)	0 (0.0%)	0 (0.0%)	0 (0.0%)	0 (0.0%)	0 (0.0%)	1 (0.1%)
Family event	0 (0.0%)	46(9.2%)	1 (0.7%)	38 (16.7%)	28 (31.5%)	13 (21.7%)	64 (20.4%)	190 (12.9%)
Job	0 (0.0%)	7 (1.4%)	0 (0.0%)	6 (2.6%)	10 (11.2%)	1 (1.7%)	13 (4.1%)	37 (2.5%)
Religious event	0 (0.0%)	3 (0.6%)	0 (0.0%)	12 (5.3%)	0 (0.0%)	5 (8.3%)	4 (1.3%)	24 (1.6%)
Other	0 (0.0%)	57 (11.4%)	9 (6.6%)	3 (1.3%)	10 (11.2%)	11 (18.3%)	73(23.2%)	163 (11.1%)
No information	106 (72.6%)	215 (43.2%)	3 (2.2%)	32 (14.0%)	5 (5.6%)	15 (25.0%)	10 (3.2%)	386 (26.2%)
NA	40 (27.4%)	159 (31.9%)	123 (90.4%)	123 (53.9%)	31(34.8%)	10 (16.7%)	119 (37.9%)	605 (41.1%)
**Witnessing polio vaccination at another location in the City**								
No	18 (12.3%)	58 (11.6%)	132 (97.1%)	87 (38.2%)	55 (61.8%)	29 (48.3%)	150 (47.8%)	529 (36.0%)
Yes	128 (87.7%)	440 (88.4%)	4 (2.9%)	141 (61.8%)	34 (38.2%)	31 (51.7%)	164 (52.2%)	942 (64.0%)
**Witnessing polio vaccination at another location outside the City**								
No	82 (56.2%)	128 (25.7%)	132 (97.1%)	140 (61.4%)	62 (69.7%)	38 (63.3%)	191 (60.8%)	773 (52.5%)
Yes	64 (43.8%)	370 (74.3%)	4 (2.9%)	88 (38.6%)	27 (30.3%)	22 (36.7%)	123 (39.2)	698 (47.5%)
**Dual Houses**								
No	128 (87.7%)	417 (83.7%)	132 (97.1%)	186 (81.6%)	83 (93.3%)	52 (86.7%)	243 (77.4%)	1241 (84.4%)
Yes	18 (12.3%)	81 (16.3%)	4 (2.9%)	42 (18.4%)	6 (6.7%)	8 (13.3%)	71 (22.6%)	230 (15.6%)
**Frequency of visit to hometown**								
Every month	4 (2.7%)	24 (4.8%)	0 (0.0%)	12 (5.3%)	1 (1.1%)	1 (1.7%)	3 (1.0%)	45 (3.1%)
Every weekend	6 (4.1%)	4 (0.8%)	0 (0.0%)	2 (0.9%)	0 (0.0%)	0 (0.0%)	1 (0.3%)	13 (0.9%)
Family events	8 (5.5%)	153 (30.7%)	2 (1.5%)	25 (11.0%)	21 (23.6%)	5 (8.3%)	99 (31.5%)	313 (21.3%)
Summer holidays	0 (0.0%)	10 (2.0%)	3 (2.2%)	0 (0.0%)	0 (0.0%)	0 (0.0%)	10 (3.2%)	23 (1.6%)
On Eid holidays	14 (9.6%)	18 (3.6%)	0 (0.0%)	19 (8.3%)	0 (0.0%)	0 (0.0%)	12 (3.8%)	63 (4.3%)
Other	32 (21.9%)	33 (6.6%)	4 (2.9%)	17 (7.5%)	4 (4.5%)	1 (1.7%)	66 (21.0%)	157 (10.7%)
No information	82 (56.2%)	175 (35.1%)	4 (2.9%)	153 (67.1%)	50 (56.2%)	2 (3.3%)	123 (39.2%)	589 (40.0%)
NA	0 (0.0%)	81 (16.3%)	123 (90.4%)	0 (0.0%)	13 (14.6%)	51(85.0%)	0 (0.0%)	268 (18.2%)
**Purpose of staying here**								
Business	0 (0.0%)	47 (9.4%)	4 (2.9%)	80 (35.1%)	48 (53.9%)	9 (15.0%)	90 (28.7%)	278 (18.9%)
Education	0 (0.0%)	1 (0.2%)	0 (0.0%)	2 (0.9%)	0 (0.0%)	0 (0.0%)	1 (0.3%)	4 (0.3%)
Family event	0 (0.0%)	5 (1.0%)	1 (0.7%)	3 (1.3%)	0 (0.0%)	1 (1.7%)	8 (2.5%)	18 (1.2%)
Job	0 (0.0%)	9 (1.8%)	0 (0.0%)	24 (10.5%)	20 (22.5%)	3 (5.0%)	39 (12.4%)	95 (6.5%)
Any other	0 (0.0%)	61 (12.2%)	11 (8.1%)	4 (1.8%)	13 (14.6%)	14 (23.3%)	118 (37.6%)	221 (15.0%)
No Information	146 (100.0%)	375 (75.3%)	120 (88.2%)	115 (50.4%)	8 (9.0%)	33 (55.0%)	58 (18.5%)	855 (58.1%)
**Source of income**								
Business	0 (0.0%)	53 (10.6%)	5 (3.7%)	0 (0.0%)	18 (20.2%)	13 (21.7%)	0 (0.0%)	89 (6.1%)
Farmer	0 (0.0%)	1 (0.2%)	88 (64.7%)	0 (0.0%)	3 (3.4%)	3 (5.0%)	0 (0.0%)	95 (6.5%)
Govt. job	0 (0.0%)	3 (0.6%)	0 (0.0%)	0 (0.0%)	5 (5.6%)	2 (3.3%)	0 (0.0%)	10 (0.7%)
Job	0 (0.0%)	0 (0.0%)	0 (0.0%)	24 (10.5%)	1 (1.1%)	0 (0.0%)	39 (12.4%)	64 (4.4%)
Private job	0 (0.0%)	12 (0.0%)	0 (0.0%)	0 (0.0%)	7 (7.9%)	8(13.3%)	0 (0.0%)	27 (1.8%)
Shopkeeper	0 (0.0%)	25 (5.0%)	3 (2.2%)	0 (0.0%)	24 (27.0%)	4 (6.7%)	0 (0.0%)	56 (3.8%)
Other	0 (0.0%)	73 (14.7%)	40 (29.4%)	0 (0.0%)	25 (28.1%)	29 (48.3%)	0 (0.0%)	167 (11.4%)
No information	146 (100.0%)	331 (66.5%)	0 (0.0%)	204 (89.5%)	6 (6.7%)	1 (1.7%)	275 (87.6%)	963 (65.5%)

Number indicates one response per household and its percentage of the total participants in that district.

**Table 3:** Comparison of characteristics of survey participants in outbreak districts in Karachi division and other divisions of Sindh, Pakistan.

Variable	Karachi	Sindh	*p*-Value
	(*N* = 1392)	(*N* = 1471)	(*N* = 2863)
**Gender**			
Female	972 (55.0%)	794 (45.0%)	<0.0001
Male	420 (38.4%)	675 (61.6%)	
**Age** (mean ± SD)	34.170 (9.5103)	35.973 (9.2622)	<0.0001
**Language**			
Balochi	81 (50.3%)	80 (49.7%)	<0.0001
Pashto	336 (86.4%)	53 (13.6%)	
Punjabi	155 (73.1%)	57 (26.9%)	
Sindhi	130 (20.6%)	501 (79.4%)	
Siraiki	118 (70.7%)	49 (29.3%)	
Urdu	376 (37.3%)	633 (62.7%)	
Others	196 (66.7%)	98 (33.3%)	
**Number of children under five years of age** (mean ± SD)	1.599 (0.9706)	1.823 (1.0955)	<0.0001
**OPV vaccination in SIA status**			
No response	15 (53.6%)	13 (46.4%)	0.003
No	67 (65.0%)	36 (35.0%)	
Yes	1310 (48.0%)	1422 (52.0%)	
**RI status**			
No	131 (39.8%)	198 (60.2%)	<0.0001
Yes partial	344 (59.1%)	238 (40.9%)	
Yes complete	917 (47.0%)	1035 (53.0%)	
**Guest arrival**			
No	334 (47.0%)	377 (53.0%)	0.312
Yes	1058 (49.2%)	1094 (50.08%)	
**Any Guest children vaccinated**			
No response	683 (52.3%)	623 (47.7%)	<0.0001
No	345 (56.5%)	266 (43.5%)	
Yes	364 (38.5%)	582 (61.5%)	
**Travel history**			
No	706 (53.8%)	606 (46.2%)	<0.0001
Yes	686 (44.2%)	865 (55.8%)	
**Purpose of travel**			
Business	5 (7.1%)	65 (92.9%)	<0.0001
Education	1 (50.0%)	1 (50.0%)	
Family event	245 (56.3%)	190 (43.7%)	
Job	50 (57.5%)	37 (42.5%)	
Religious event	38 (61.3%)	24 (38.7%)	
Other	121 (42.6%)	163 (57.4%)	
No information	226 (36.9%)	386 (63.1%)	
NA	706 (53.9%)	605 (46.1%)	
**Witnessing polio vaccination at another location in the City**			
No	689 (56.6%)	529 (43.4%)	<0.0001
Yes	703 (42.7%)	942 (57.3%)	
**Witnessing polio vaccination at another location outside the City**			
No	839 (52.0%)	773 (48.0%)	<0.0001
Yes	553 (44.2%)	698 (55.8%)	
**Dual Houses**			
No	1136 (47.8%)	1241 (52.2%)	0.050
Yes	256 (52.7%)	230 (47.3%)	
**Frequency of visit to hometown**			
Every month	44 (49.4%)	45 (50.6%)	<0.0001
Every weekend	26 (66.7%)	13 (33.3%)	
Family events	365 (53.8%)	313 (46.2%)	
Summer holidays	32 (58.2%)	23 (41.8%)	
On Eid holidays	67 (51.5%)	63 (48.5%)	
Other	301 (65.7%)	157 (34.3%)	
No information	557 (48.6%)	589 (51.4%)	
NA	0 (0%)	268 (100.0%)	
**Purpose of staying here**			
Business	183 (39.7%)	278 (60.3%)	<0.0001
Education	23 (85.2%)	4 (14.8%)	
Family event	22 (55.0%)	18 (45.0%)	
Job	510 (84.3%)	95 (15.7%)	
Other	69 (23.8%)	221 (76.2%)	
No information	585 (40.6%)	855 (59.4%)	
**Source of income**			
Business	83 (48.3%)	89 (51.7%)	<0.0001
Farmer	12 (11.2%)	95 (88.8%)	
Govt. job	20 (66.7%)	10 (33.3%)	
Job	257 (80.1%)	64 (19.9%)	
Private job	238 (89.8%)	27 (10.2%)	
Shopkeeper	24 (30.0%)	56 (70.0%)	
Other	75 (31.0%)	167 (69.0%)	
No information	683 (41.5%)	963 (58.5%)	

## Data Availability

The datasets used and analyzed during the current study are available at the EOC Sindh (sindh.eoc@gmail.com) upon a reasonable request.

## Supplementary Materials

The following supporting information can be downloaded at: https://www.mdpi.com/article/10.3390/vaccines13101016/s1, Supplementary Table S1. Genetic linkages of different wild poliovirus type 1 isolates were reported in Sindh province from April to August 2024.; Supplementary Material S2. Questionnaire used in study “Redefining High Risk and Mobile Population in Pakistan Polio Eradication Program; 2024”.
