# Supplementary material for: Redefining High-Risk and Mobile Population in Pakistan Polio Eradication Program; 2024
Source: Vaccines (Basel). 2025 Sep 29;13(10):1016. doi: 10.3390/vaccines13101016 (PMC12567959; doi:10.3390/vaccines13101016)
Supplement: Supplementary file 1 [file vaccines-13-01016-s001.zip › vaccines-3556358-supplementary.pdf]

vaccines

MDPI

Article

Redefining High Risk and Mobile Population in Pakistan Polio Eradication Program; 2024

Supplementary Table S1. Genetic linkages of different wild poliovirus type 1 isolates were reported in Sindh province from April to August 2024.

Months	District	Participants Number	Reason	Location of virus with closest detected genetic Linkage
April	Badin	146	ES WPV1 Positive	Haji Mureed Goth Central Karachi
	Central, Khi	160	ES WPV1 Positive	Haji Mureed Goth Central Karachi
	Hyderabad	164	ES WPV1 Positive	Haji Mureed Goth Central Karachi
May	Central, Khi	318	ES WPV1 Positive	Haji Mureed Goth Central Karachi
	Keamari, Khi	45	ES WPV1 Positive	Orangi Nalla Keamari Karachi
	Mirpurkhas	228	ES WPV1 Positive	Orangi Nalla Keamari Karachi
	Sukkur	314	ES WPV1 Positive	Haji Mureed Goth Central Karachi
	West, Khi	225	ES WPV1 Positive	Orangi Nalla Keamari Karachi
June	Malir, Khi	240	Shikarpur WPV1 human case Travel history of Malir Karachi	Hijrat Colony South Karachi
July	Keamari, Khi	186	Keamari WPV1 case	Orangi Nalla Keamari Karachi
	Kambar	136	ES WPV1 Positive	Haji Mureed Goth Central Karachi
	South, Khi	60	Keamari WPV1 human case Travel history of South Karachi	Orangi Nalla Keamari Karachi
August	Hyderabad	160	Hyderabad WPV1 human case	ES Hyderabad
	Shaheed Benazirabad	90	ES WPV1 Positive	ES Okara, Punjab
	Sujawal	60	ES WPV1 Positive	ES Hyderabad

ES = environmental surveillance
WPV1 = wild poliovirus type 1

Supplementary Material S2

Questionnaire

Questionnaire

High Risk and Mobile Population – Movement Tracking Survey

District -- **UC** --
Area name -- **Area Supervisor name** ------------------------------------
Team name/number ---------------------------------- **Household number** ---------------------------------------
----

S. No.	Questions	Response
1	Age of the survey participant (specify age in years)	Specify -------------------
2	Gender of the survey participant	0. Female<br>1. Male
3	The language household members speak at home.<br>(Pashto, Balochi, Afghani/Dari, Sindhi, Urdu, Punjabi, must specify if other)	Specify -------------------
4	The tribe household members belong to;<br>(Aka Khel, Suleman Khel, Kharoti, Uzbek, Tajik, Hazara, Kakar, must specify if other)	Specify -------------------
5	How many are children under 5 years of age in this family?	No. -----------------------
6	How many are children under 2 years of age in this family?	No. -----------------------
7	Does your child get all routine immunization vaccines?	0. No,<br>1. Yes, some vaccines,<br>2. Yes, all vaccines
8	Has any polio worker visited your house recently?	0. No<br>1. Yes
8.1	If yes, then specify the month when they visited	1. Jan<br>2. Feb<br>3. Mar<br>4. Apr<br>5. May
9	Has your child received the Polio (OPV) vaccination?	0. No<br>1. Yes

9.1	If a child received no OPV vaccine, then specify the reason.	1. NA,<br>2. Refusal<br>3. Newborn<br>4. No team visit<br>5. Sick<br>6. Other
9.2	If yes (OPV received) then specify the month of the last vaccination	1. Jan<br>2. Feb<br>3. Mar<br>4. Apr<br>5. May
10	Have our teams asked about any guests who arrived at your home within the last 3 months?	0. No<br>1. Yes
10.1	If yes, specify the district from where the guest arrived	----------------------------
10.2	Has our team vaccinated any guest children at your home?	0. No (> 5 years old guest only)<br>1. No (<5 years child guest present but not vaccinated)<br>2. Yes
11	Did the polio team ask about your or any family member's travel outside the district/ city within the last 3 months?<br>(Or do any family members travel outside the district/ city?)	0. No<br>1. Yes
11.1	If yes, specify the district/travel destination	----------------------------
11.2	What was the purpose of travel?	0. Job<br>1. Education<br>2. Business<br>3. Family event<br>4. Any Other
12	Have you observed polio team visits at the house of any of your relatives **within your district/ city** in the last 3 months?	0. No<br>1. Yes
12.1	If yes, specify the area/district name	----------------------------
13	Have you observed polio team visits at the house of any of your relatives **outside your district/city** in the last 3 months?	0. No<br>1. Yes

13.1	If yes, specify the city/district name.<br>(Mirpurkhas, Badin, Hyderabad, Central Karachi, Keamari Karachi, West Karachi etc.,)	-----------------------------
14	Since how long have you been living in this area?<br>Must specify the duration in months or years	Specify -------------------
14.1	Where you were living before coming to this area/ district (district of origin)?<br>(Mirpurkhas, Badin, Hyderabad, Karachi etc.,)	Specify -------------------
15	Do you have a house/residence in any other area /district? (Dual house)	0. No<br>1. Yes
15.1	If yes, please specify the area/district<br>(Mirpurkhas, Badin, Hyderabad, Karachi etc.,)	-----------------------------
16	How frequently do you visit your hometown?	1. Every weekend<br>2. Every month<br>3. Family Events<br>4. On Eid holidays<br>5. Summer holidays<br>6. Other
17	What is the purpose of staying here?	0. Job<br>1. Education<br>2. Business<br>3. Any Other
18	What is the source of income of the head of the household?<br>(Job, Business, farmer, teacher, shopkeeper,<br>Other must specify)	Specify ------------------
19	Do you have any observations to share about polio teams?	Specify ------------------
20	Do you have any suggestions to improve the performance of the polio team/ polio program?	Specify -------------------

We thank you for your time and feedback.
